# Codevelopment, Implementation, and Evaluation of an AI-powered Conversational Interface for Tobacco Cessation: Protocol for a Single-Group Pre-Post Study

**DOI:** 10.2196/82264

**Published:** 2026-10-07

**Authors:** Monika Arora, Rajmohan Panda, Shalini Bassi, Kshitij Jadhav, Prabhat Chand, Monirujjaman Biswas, Mohai Menul Biswas, Tina Rawal, Navpreet Kaur, Sharmila Pimple, Srabana Misra Bhagabaty

**Affiliations:** 1 Public Health Foundation of India New Delhi India; 2 Koita Centre for Digital Health Indian Institute of Technology Bombay Mumbai India; 3 Department of Psychiatry, Centre for Addiction Medicine & NIMHANS Digital Academy National Institute of Mental Health and Neuro Sciences Bengaluru India; 4 Department of Preventive Oncology, Centre for Cancer Epidemiology Tata Memorial Centre Mumbai India; 5 Department of Preventive Oncology Dr. Bhubaneswar Borooah Cancer Institute Guwahati India

**Keywords:** tobacco cessation, artificial intelligence, conversational interface, mobile app, smokeless tobacco, India

## Abstract

**Background:**

Despite several efforts to expand tobacco cessation services through tobacco cessation centers (TCCs) and quit lines, key operational challenges still persist. Recently, AI-based digital interventions have shown promise globally for smoking cessation; however, they remain underused in tobacco cessation strategy in the Indian context.

**Objective:**

This study aimed to (1) codevelop the conversational interface with beneficiaries and cessation providers; (2) assess its feasibility, acceptability, usability, and user engagement; and (3) evaluate its effectiveness in promoting attempts and intention to quit tobacco.

**Methods:**

The study consists of three phases: (1) codeveloping the comprehensive AI-powered Conversational Interface to Quit Tobacco (CARE) conversational interface through in-depth interviews with tobacco users, counselors, and health care professionals; (2) feasibility testing with tobacco users to assess engagement, usability, and acceptability; and (3) 6-month effectiveness testing using pre-post surveys. The CARE conversational interface will be developed using a retrieval-augmented generation–based large language model by the Indian Institute of Technology (Bombay, Maharashtra, India), delivering personalized, multilingual cessation support via a chatbot integrated into a mobile app. Key evaluation measures will use validated tools such as the Fagerström Test for Nicotine Dependence, Smoking Self-Efficacy Questionnaire, Decisional Balance Scale, and a Knowledge Score Questionnaire. Data will be analyzed using mixed methods, including thematic analysis for qualitative data and descriptive statistics and a multivariate logistic regression for quantitative data.

**Results:**

The CARE study was funded in March 2025 and is being implemented across 3 TCCs. Preparatory activities, including tool development, site engagement, ethics approval, and trial registration (CTRI/2024/11/076916), were completed in 2025. Phase 1 (codevelopment) is scheduled from February to August 2026, followed by phase 2 (feasibility testing) from September 2026 to August 2027, and phase 3 (effectiveness assessment) from September to November 2027. Data analyses are expected to be completed by late 2027, with key findings on feasibility, acceptability, usability, user engagement, and preliminary cessation outcomes targeted for peer-reviewed publication from 2027 onward.

**Conclusions:**

The CARE study will attempt to introduce a novel, culturally tailored conversational interface targeting both smokers and smokeless tobacco users in India by integrating AI-based solutions as an adjunct to conventional counseling at the TCCs. Given the single-group pre-post design without a control arm, feasibility and effectiveness findings will be interpreted as preliminary and hypothesis-generating rather than causal evidence of intervention impact. The intervention approach of codevelopment, strengthening capacity in evidence-based content, and enabling a multilingual conversation interface is expected to enhance tobacco user engagement and improve cessation outcomes. Findings from this pilot will inform a future randomized controlled trial and provide evidence for the potential integration of the CARE interface into India’s tobacco cessation platforms, offering a low-cost, high-impact solution for India and other low- and middle-income countries that face similar challenges.

**Trial Registration:**

Clinical Trials Registry–India CTRI/2024/11/076916; https://tinyurl.com/3vfs5d8s

**International Registered Report Identifier (IRRID):**

PRR1-10.2196/82264

## Introduction

In India, tobacco use poses a significant health risk, contributing to 1.35 million deaths annually [1], with smokeless tobacco (SLT) being the most prevalent form of tobacco use [2]. In 2020, tobacco-related malignancies accounted for 27% of all reported cancer cases in India [3-5]. A meta-analysis of studies conducted in India between 2010 and 2022 found that the prevalence of tobacco use was highest in the East region (55.4%), followed by the Northeast region with 51.8% tobacco consumption [4]. Evidence from the 2017-2018 Longitudinal Aging Study in India (LASI) indicated that geographical region strongly influences tobacco use among middle- and old-aged women (45 years and above), with the highest odds observed in the Northeast region (adjusted odds ratio [AOR] 5.260, 95% CI 4.469-6.191), followed by the East (AOR 2.211, 95% CI 1.930-2.533) and the West (AOR 1. 1.977, 95% CI 1.677-2.332) regions of India [6].

Tobacco cessation efforts in India began in 2002 with 19 tobacco cessation centers (TCCs) in tertiary care [7], which expanded to over 500 TCCs under the National Tobacco Control Programme (2007-2008) [8]. These efforts were further extended in 2016 by the establishment of the National Tobacco Quitline Services (NTQLs) [9], regional satellite centers in 2018 [10], mCessation [11], de-addiction services, and the establishment of TCCs in dental institutes under the National Oral Health Programme [12]. Despite these efforts, significant challenges persist, including counselor unavailability, inadequate counseling facilities, lack of information on quitting [13], high loss to follow-up [14], and infrastructure constraints such as poor internet connectivity and frequent call failures [15]. Additional barriers such as accessibility of services, stigma, time constraints, and low user motivation continue to hinder service uptake. A significant missed opportunity occurs when motivated tobacco users call cessation services but find counselors unavailable, limiting timely support for quit attempts. These operational challenges and implementation gaps, both in demand and supply, contribute to suboptimal use of tobacco cessation services.

COVID-19 lockdowns presented an unintended but valuable cessation opportunity, as the unavailability of tobacco products forced many users into abrupt abstinence. Studies reported that restrictions on tobacco sales during the lockdown led to reductions in tobacco use, quit attempts, and even quitting among substantial proportions of both smokers and SLT users [16]. However, the COVID-19 pandemic also exposed the need for continuous support mechanisms, as many users relapsed when tobacco products became available again and when cessation counseling services were inaccessible.

The Global Adult Tobacco Survey-2 (GATS-2, 2016-2017) underscored a substantial gap between quit intentions and the use of cessation support in India. Although more than half of current smokers (55.4%) and current SLT users (49.6%) expressed a desire to quit, only a small proportion accessed evidence-based assistance, including pharmacotherapy (4.1% of smokers; 3.2% of SLT users) or counseling support (8.6% of smokers and 7.3% of SLT users) [2]. Against this backdrop, the target population for the CARE study is current tobacco users (both smokers and SLT), who are actively seeking services at TCCs. This group represents a motivated, high-need subpopulation within India’s broader tobacco epidemic, where overall tobacco prevalence remains high at 28.6% (10.7% smokers and 21.4% SLT users) [2,8]. TCC attendees are particularly well suited for adjunct digital tools such as CARE, as they are already engaged with cessation efforts yet often face structural constraints, including limited counselor availability [4]. Focusing on this population also supports scalability across diverse patterns of tobacco use, including the predominance of SLT in India.

The use of technologies and digital platforms has expanded several opportunities for delivering behavioral support for tobacco cessation. According to the Mohr model of supportive accountability, human-guided digital health interventions can enhance user engagement by fostering personal accountability. Recently, AI advancements have been increasingly investigated to simulate human care in order to bridge the gap between digital interventions and professional, customized support [17].

Research from other countries revealed that innovative solutions, such as mobile-and web-based [18-22] and AI-driven interventions, particularly conversational AI interventions (chatbots), hold promise in supporting tobacco cessation outcomes [23]. Conversational AI, typically referred to as a chatbot or a dialog system, enables 2-way communication with users via text and/or audio without human input by using natural language processing and machine learning algorithms. Research has shown that participants in the conversational AI–enhanced intervention were significantly more likely to quit smoking at 6-month follow-up compared to control group participants [24]. However, limitations such as restricted vocabulary, voice recognition inaccuracies, and dissatisfaction from the absence of a personal connection highlighted the need for further refinement [25].

Despite considerable interest in AI-based cessation interventions, India lacks rigorously tested, evidence-based, AI-powered cessation tools, codeveloped with tobacco users and cessation providers. Many existing apps fall short of clinical guidelines for tobacco cessation [26,27], offer limited engagement, are inaccessible due to high costs [28], are available in English, and have a sole focus on smoking [28]. The evidence of long-term continuous abstinence using apps or AI-based cessation programs is limited in the Indian context. A smartphone-based app for patients with alcohol dependence showed a significant decrease in app usage after the first week [29], demonstrating the need for empirically grounded AI-based cessation solutions that are codeveloped with users seeking cessation and counselors offering cessation services.

To address these gaps, the study proposes the codevelopment, implementation, and evaluation of a Comprehensive AI-powered Conversational Interface to Quit Tobacco (CARE), a multilingual, user-informed conversational AI platform to improve cessation among tobacco users. This paper presents the protocol for codeveloping, implementing, and evaluating CARE, outlining the planned methodology for its development, feasibility testing, and evaluation phases. Therefore, the study has the following objectives:

1. To codevelop an AI-based conversational interface using large language models (LLMs) to strengthen tobacco cessation behaviors at TCCs
2. To assess the feasibility, acceptability, usability, and user engagement of the proposed AI-based conversational interface for tobacco users at TCCs
3. To evaluate a generative AI–based conversational interface to facilitate successful quit attempts and increase intention to quit among tobacco users at TCCs

## Methods

### Study Team and Collaborators

The study is being developed and implemented through a collaborative effort by the Public Health Foundation of India (PHFI) in partnership with key partners, including Tata Memorial Centre (TMC; Mumbai, Maharashtra), National Institute of Mental Health and Neurosciences (NIMHANS; Bengaluru, Karnataka), Dr. B. Borooah Cancer Institute (BBCI; Guwahati, Assam), and Indian Institute of Technology Bombay (IIT-B). All partner organizations actively participated in the initial planning phase and contributed to the development of the study protocol, study documents, and data collection tools. The site partners will play a crucial role in participant recruitment, leveraging their contextual knowledge and on-ground experience, which will be essential throughout the implementation of the CARE study.

### Study Setting

The study is being conducted across 3 geographically and demographically diverse TCCs in India: TMC, BBCI, and NIMHANS. Tobacco users currently availing services from these TCCs form the primary study participants. Participant recruitment in phases 1, 2, and 3 will involve distinct populations of tobacco users. Eligibility criteria are listed in Textbox 1.

**Eligibility criteria for study participants.**

**Inclusion criteria**

- Adults aged 18 years and above
- Willing to provide informed consent
- Using any form of tobacco (smoking, smokeless, or dual use) for the past 6 months
- Currently availing cessation services at the tobacco cessation center (TCC)
- Interested in quitting tobacco
- Ownership of an Android (Google, Inc) or iOS (Apple, Inc) smartphone (Android 11+or iOS 13+)
- Basic digital literacy
- Access to permanent contact information

**Exclusion criteria**

- Individuals currently enrolled in another digital tobacco cessation program
- Individuals who decline to provide informed consent
- Pregnant women with complications
- Individuals with severe mental health conditions

### Study Design

The study adopts a single-group pre-post design, implemented in 3 sequential phases (Figure 1) without a control group. Phase 1 involves codevelopment of the CARE conversational interface using insights from qualitative in-depth interviews (IDIs) with tobacco users, tobacco cessation counselors, and health care professionals (HCPs), and established behavioral theories and evidence-based cessation practices [30]. Phase 2 assesses feasibility, usability, acceptability, and user engagement with the CARE conversational interface that will be assessed through structured quantitative surveys and qualitative interviews with tobacco users. Phase 3 evaluates the effectiveness of the CARE conversational interface through a pre-post survey to assess its impact on quit attempts, intention to quit, and tobacco-related behavioral outcomes. As the study uses a single-group pre-post design without a concurrent control group, observed changes in quit attempts and intention to quit will be interpreted as within-participant changes over time. The design does not permit definitive attribution of effects solely to the CARE intervention. The findings will therefore be interpreted cautiously, with consideration given to potential confounding factors, secular trends, and other external influences that may contribute to observed changes over time.

**Figure 1:**
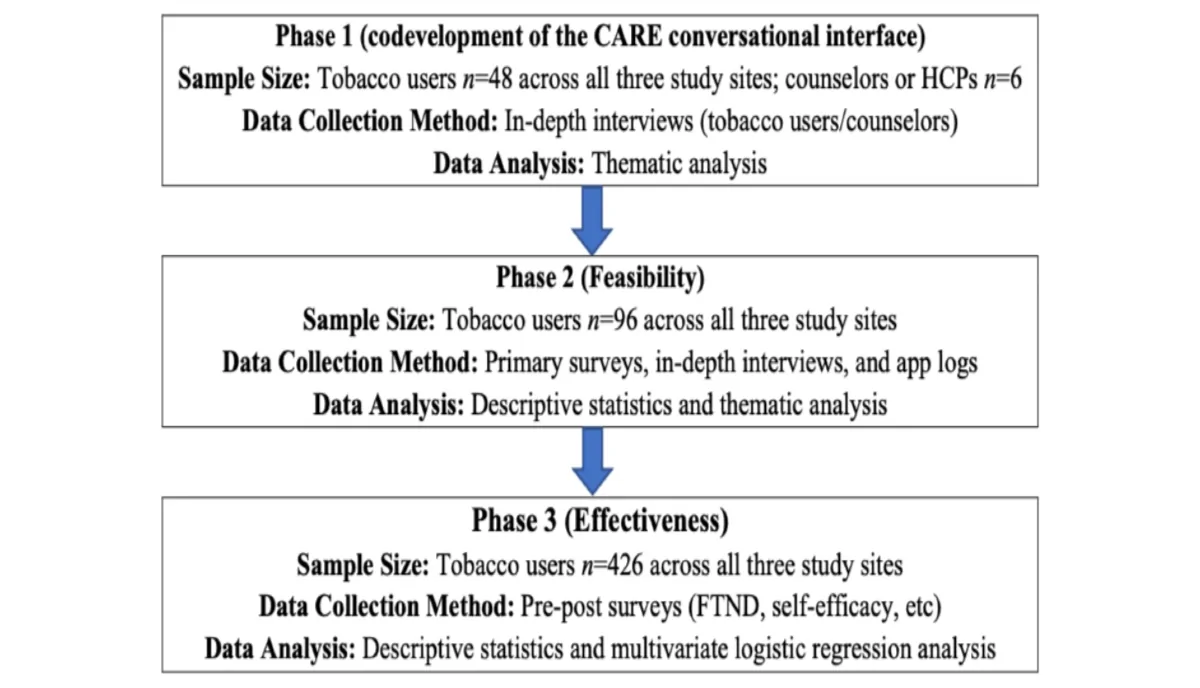
Phased development, implementation, and evaluation of CARE. CARE: Comprehensive AI-powered Conversational Interface to Quit Tobacco; FTND: Fagerström Test for Nicotine Dependence ; HCP: health care professional.

The study will be guided by the Designing, Developing, Evaluating, and Implementing a Smartphone-Delivered, Rule-Based Conversational Agent (DISCOVER) conceptual framework for user-centered digital health interventions [31]. The framework provides a structured, theory-informed approach for the codevelopment, implementation, and evaluation of CARE. The framework will ensure the intervention is contextually relevant, user-centered, and grounded in behavioral science. The framework structures the process so that early phases focus on defining the problem, understanding the context, and identifying user needs rather than predefined solutions. Subsequent phases prioritize iterative co-design, using input from tobacco users and stakeholders’ feedback to refine content, language, behavior change techniques, and delivery modalities, while later phases align evaluation with key domains (feasibility, usability, engagement, and effectiveness) with measurable indicators to ensure that the intervention is acceptable, usable, and implementable in real-world settings.

### CARE Conversational Interface

The CARE conversational interface will include several core modules designed to provide structured, personalized, and scalable support for tobacco users (Figure 2). Key features include:

**Figure 2:**
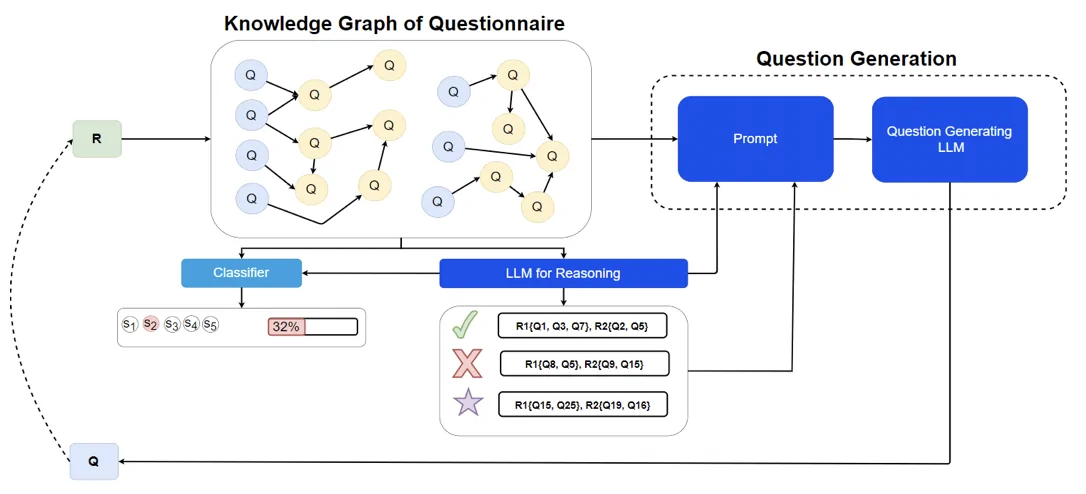
Architecture diagram of CARE app. CARE: Comprehensive AI-powered Conversational Interface to Quit Tobacco; LLM: large language model.

1. Dedicated support: this allows the tobacco user to interact with an AI-powered chatbot, with an option to escalate to a tobacco cessation specialist when needed.
2. Craving monitoring: this allows tobacco users to track and manage cravings (strong desire toward tobacco use).
3. Reward system: this offers reinforcement through the display of reward badges as users achieve milestones.
4. Main dashboard: this provides users with graphs illustrating health gains and monetary savings accrued from quitting tobacco.
5. Motivational messages: messages will be designed based on the findings from semistructured interviews with tobacco users and cessation experts. The messages for each topic (IDIs, readiness to quit, dealing with cravings, and relapse prevention) will be developed and validated by the cessation experts.

The conversational interface will use algorithms that can adapt to the constantly evolving needs and interests of tobacco users and offer personalized recommendations, leveraging so-called “collective intelligence.”

### The Components of CARE Conversational Interface

The CARE conversational interface primarily comprises 2 key components:

1. CARE app: The CARE app serves as the user interface for delivering AI-enhanced, personalized counseling support to tobacco users through accessible platforms such as WhatsApp (Meta Platforms, Inc.). It will provide targeted support to both smokers and SLT users, addressing the unique needs of urban and rural populations. Additional features include multilingual support to cater to diverse populations, customized quit planning, real-time contextual messaging, 24/7 availability, addressing time limitations of traditional TCCs, consistent nonjudgmental support that minimizes stigma and interpersonal variability in counseling, and interactive chatbot functionality to answer user queries and reinforce engagement. It aims to overcome interpersonal variability in counselor skills, take a proactive approach through timely notifications, and reduce stigma often experienced by tobacco users when interacting with counselors.
2. AI-based messaging engine: The messaging engine integrated into the CARE app will offer a chatbot, enabling personalized, automated, and continuous counseling support using advanced AI techniques. It will be built on a retrieval-augmented generation (RAG)–based large language model, coupled with multilingual speech recognition to deliver culturally relevant, conversational, and tailored cessation guidance (Figure 3). The RAG-based LLM is grounded in a curated knowledge repository comprising peer-reviewed literature, national tobacco cessation guidelines, training manuals, operational protocols, and routine clinic workflows, informed by established behavior change models. The system uses an ontology-driven hybrid RAG pipeline with three stages: (1) auto-ontology generation to extract clinically relevant concepts and relationships; (2) ontology-aware semantic chunking of source documents; and (3) hybrid retrieval combining keyword search, vector similarity, and knowledge graph–based concept matching with reranking for stage-appropriate advice. All participants will receive standardized, stage-matched, evidence-based counseling delivered through a fixed RAG-based framework with version-controlled prompts and retrieval corpus to ensure consistency across languages and study phases. Bias and hallucination risks are mitigated by restricting generation to retrieved evidence-based content, using structured ontology constraints. In addition, predefined risk and sensitivity triggers are incorporated at runtime to suppress or refuse unsafe responses and enable escalation to trained human counselors, when necessary, with interaction logs periodically reviewed to identify and correct potentially harmful outputs.
3. The development of the messaging engine will draw upon the stages of change of the Transtheoretical Model (TTM) [32], components of relapse prevention based on cognitive behavior theory, an adapted version of the AI chatbot behavior change model [33], previously published scientific research of the investigators [34], clinical guidelines for treating tobacco use and dependence [35], the 5 A’s model (Ask, Advise, Assess, Assist, and Arrange), and evidence-based tobacco cessation algorithms used in clinical practice [36]. The key features and functionalities (Table 1) of the CARE interface will be informed by insights gathered during the co-design phase. The design approach will prioritize user accessibility, multilingual support, and a user-friendly interface to ensure broad adoption, especially across diverse user groups. In parallel, the accuracy and cultural appropriateness of the speech-to-text (automatic speech recognition; ASR), translation, and text-to-speech (TTS) components will be systematically evaluated through a combination of domain-specific testing and benchmark validation. ASR performance will be assessed using curated speech datasets representing both controlled and real-world conditions, with balanced gender representation, and benchmarked against multiple state-of-the-art models using standard metrics such as word error rate (WER). Speech output will leverage open-source state-of-the-art TTS models selected for natural intonation, low latency, and robust handling of code-mixed inputs to support context-appropriate counseling delivery. Translation quality and multilingual robustness will be further validated using established open-source benchmark datasets, including IndicVoices [37] and BhasaAnuvaad [38], complemented by iterative expert review to ensure linguistic accuracy and cultural appropriateness across Indian languages.

**Figure 3:**
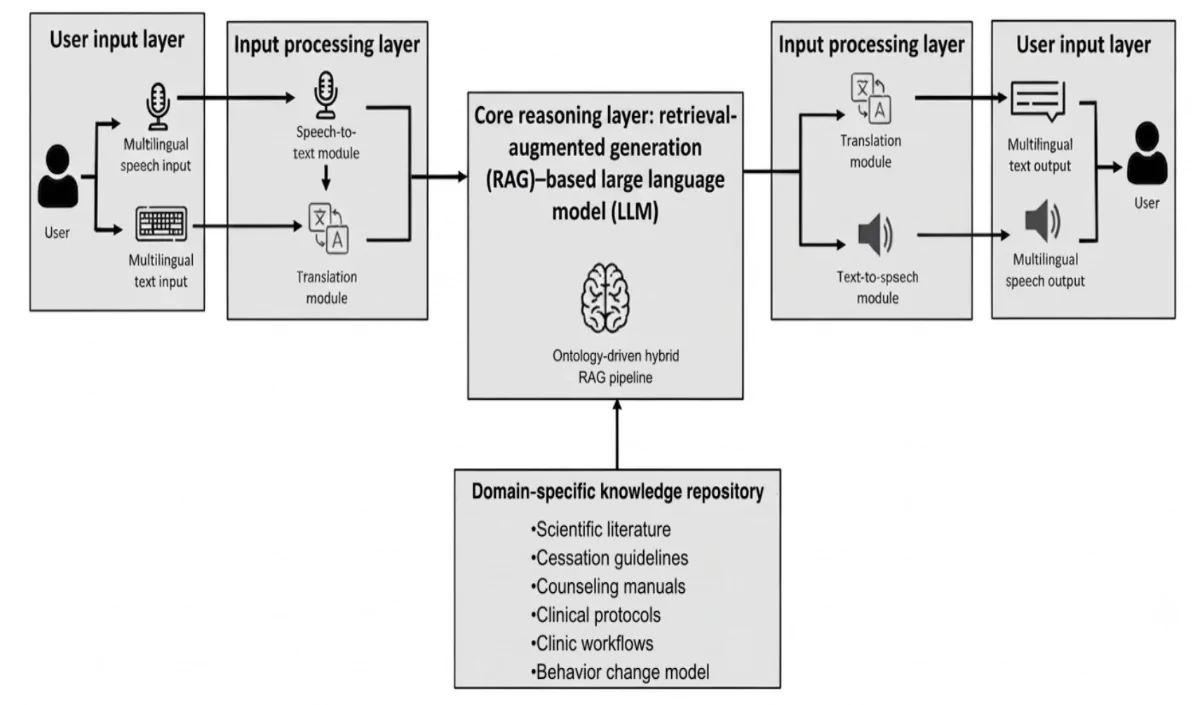
AI-based chat engine in the CARE app. CARE: Comprehensive AI-powered Conversational Interface to Quit Tobacco.

**Table 1:** Key features of the Conversational Interface to Quit Tobacco app.

Features	Description
Integrated AI-based messaging chatbot	A user-friendly chatbot to support both voice- and text-based interactions. It will deliver personalized, evidence-based advice tailored to the user’s quit stage and preferences. The chatbot will incorporate self-learning algorithms to adapt to the evolving needs of users, leveraging collective intelligence to improve recommendations continuously.
Dedicated support	An option for users to connect with a tobacco cessation specialist for additional help when needed.
Quit plan management and tracking	Users can set quit goals, monitor their progress, and adjust plans as needed.
Notifications and reminders	Timely prompts and motivational nudges to keep users engaged and on track with their quit plans.
Gamification elements	Engaging features such as progress badges and rewards to enhance motivation and user retention.
Peer support group	Enabling users to connect with others on similar journeys for mutual encouragement and accountability.
Follow-up management	Structured follow-up modules to monitor progress, identify setbacks, and provide timely support.
Craving monitoring	Tools to help users log and manage cravings, enabling self-awareness and behavioral adjustment.
Reward section	Reinforcement through virtual badges and tokens acknowledging milestones and sustained efforts.
Main dashboard	A visual interface displaying health benefits achieved and financial savings from quitting tobacco to reinforce motivation.
Motivational messages	Carefully crafted messages based on semistructured interviews with tobacco users and cessation experts from TCCs^a^. These will be topic-specific, validated by experts, and tailored to address user-specific challenges and motivators.

^a^TCC: tobacco cessation center.

### Intervention Package

All enrolled participants will receive a standardized core intervention package comprising (1) onboarding and informed consent within the app, (2) baseline stage-of-change assessment, (3) personalized quit plan generation, (4) access to AI-based conversational counseling (text and/or voice), (5) scheduled motivational messages, (6) craving monitoring functionality, and (7) access to escalation pathways to human counselors when indicated. These components constitute the minimum intervention exposure delivered uniformly across all participants during phases 2 and 3.

The AI messaging engine operates on a centralized, curated knowledge repository derived from national tobacco cessation guidelines, peer-reviewed literature, and expert-validated behavioral modules. All counseling content is first developed in a master reference language and then translated using validated translation pipelines. Outputs in regional languages undergo expert review to ensure conceptual equivalence, cultural appropriateness, and alignment with evidence-based cessation principles. The RAG framework ensures that responses are constrained to this approved knowledge base, thereby minimizing variability across languages.

The CARE conversational model, including architecture, model weights, system prompts, and the retrieval corpus, will be version-locked at the start of phase 2. No substantive updates to the model parameters, knowledge repository, or prompting logic will be implemented during phases 2 and 3 unless required for safety reasons. Any modifications will be formally documented, reviewed by the study’s technical oversight team, and, if necessary, submitted for ethics review prior to deployment. This ensures intervention consistency throughout the evaluation period (Table 1).

### Phase 1: Development of CARE Conversational Interface

#### Study Population

To inform the development of the CARE conversational interface, a purposive sample of tobacco users (n=48) with maximum variation across the type of tobacco use (smoking, SLT, and dual users), socioeconomic status (rural and urban), and sex (male and female) will be recruited across the 3 TCCs (n=16 per study site). A sample of counselors or HCPs (n=6; 2 per study site) will also be recruited.

Patient profiling will involve the collection of comprehensive demographic information, including age, gender, education, occupation, socioeconomic status, and geographic location, to understand the population characteristics. Detailed tobacco use history will be documented, covering types of tobacco consumed (smoking and smokeless), usage, frequency and duration, age of initiation, previous quit attempts, and cessation methods used. Additionally, participants’ ease and access to technology and digital literacy (including mobile phone ownership, smartphone usage, and familiarity with digital health platforms) will be assessed to deliver AI-driven intervention effectively.

#### Data Collection

Adopting a cocreation approach, IDIs with tobacco users, counselors, and HCPs will be conducted to comprehensively understand both facilitators and barriers faced by tobacco users in quitting and counselors in offering tobacco cessation services. Separate semistructured IDI guides will be developed for tobacco users and counselors, informed by the existing literature and study objectives. These interviews will generate insights for the development of the CARE conversational interface, building on prior health interventions [31,39-43]. The IDIs will be conducted in person at the TCCs by trained qualitative researchers recruited at 3 study sites. TCCs will approach the potential participants and enroll them upon providing informed consent. All interviews will be audio-recorded following participants’ consent, while detailed notes will be taken during the sessions to capture nonverbal communication and contextual cues. Thematic analysis will be done after the data collection.

### Phase 2: Feasibility Testing

#### Study Population

A random sample of tobacco users (n=96; 32 per study site) will be selected from TCCs to evaluate the feasibility, acceptability, usability, and user engagement with the CARE intervention. The sample size is determined based on the study’s exploratory nature.

#### Data Collection

Following 3 months of CARE intervention use, these outcomes will be evaluated across three key dimensions: actual app usage patterns captured by the CARE interface, direct user feedback through an adapted version of a structured Attitudinal Survey [44], and structured qualitative insights gathered from semistructured IDIs. This will be ensured across all 3 study sites using predefined, standardized, validated tools, guides, and training packages.

#### Participants Engagement Safeguards

To mitigate risks of low app engagement and ensure robust data collection, the protocol includes study team–led procedural steps independent of app features. No strict minimum usage threshold will be imposed, as even low engagement provides valuable real-world feasibility data, but weekly aggregated app metrics (eg, sessions opened and interaction time <5 minutes/week) will be monitored via a secure dashboard during phases 2 and 3 [45]. If <50% (phase 2: <48/96; phase 3: <213/426) of participants show minimal use (defined as <3 sessions in first month) by week 4, site coordinators will conduct protocolized phone check-ins (separate from counseling) to troubleshoot barriers (eg, technical issues and digital literacy) and reinforce optional participation, documented in logs [46,47]. For analysis, engagement will be reported in a descriptive way (mean, proportions, and medians); if median sessions are <3 or if attrition is >30% due to nonuse, feasibility will be deemed low, primary effectiveness analyses will be limited to engaged users, and recruitment will be expanded by 20%. Low engagement scenarios will be transparently discussed as implementation barriers in reporting [45,48].

A structured attitudinal survey will be administered to assess feasibility (extent to which the CARE app performs its intended functions, its practicality, utility, and suitability for everyday use), acceptability (users’ affective attitudes, comfort level, and perceived advantage of using the CARE app), usability (users’ perception of the support or help provided by the CARE app), and user engagement (will be assessed using usage metrics including app access frequency, interaction time, extent of the dialog, initiated conversations, return users, and session duration). A survey will include a validated Likert scale to evaluate users’ perceived usability, content clarity, satisfaction, and engagement.

IDIs will be conducted with 96 tobacco users by a team of trained qualitative researchers from social science backgrounds in the regional language. A participant information sheet before participation will provide essential details about the study, including its purposes, procedures, potential risks and benefits, privacy and confidentiality measures, and the participant’s rights, ensuring informed consent. The IDIs will be conducted using a semistructured interview guide, audio-recorded, comprising open-ended questions. These will explore participants’ quit attempts, methods used for quitting, motivation to quit, and factors influencing both successful and unsuccessful quit attempts. The interviews will also examine participants’ perceptions and attitudes toward the CARE app, initial and enduring challenges, and their overall acceptability and experience with the CARE app (Multimedia Appendix 1). The data collection will continue until data saturation is achieved, following the principle of informational redundancy [49].

Actual app usage patterns will be compiled by the CARE app, including metrics such as number of times the CARE app is opened, time spent interacting, extent of dialog, and average session duration.

### Phase 3: Evaluating Effectiveness

#### Study Population

A total of 426 participants will be recruited across the 3 study sites (142 participants per study site) to evaluate the effectiveness of the CARE conversational interface in improving quit attempts and intentions to quit.

#### Sample Size

The sample size was determined using G*Power software (Faul, Erdfelder, Lang, and Buchner), assuming a small, expected effect size (d=0.22), a significance level α of .05, and 90% statistical power. Accounting for an intracluster correlation coefficient of 0.01 and a design effect of 1.54, the required sample size was estimated at 339 participants to maintain robust statistical power and accommodate center similarities. An attrition rate of 20% will be factored in to guarantee resilient statistical power, resulting in a final sample size of 426 participants [50,51].

#### Data Collection

Participants will complete a baseline survey assessing their tobacco use status, intention to quit, and key psychosocial variables. The same measures will be reassessed 6 months after the participants have engaged with the CARE app. The post-CARE survey will evaluate cognitive and behavioral determinants of cessation outcomes, including tobacco cessation knowledge (hazard score), attitudes toward tobacco use, and readiness to quit (stages of change). Trained researchers will administer an adapted set of validated questionnaires, including questions about demographics, Fagerstrom Test for Nicotine Dependence [52], Knowledge Score questionnaire [53], Process of Tobacco Cessation survey [32], Smoking Self-Efficacy questionnaire [54], and Decisional Balance Scale [55]. These have been previously used and validated in Indian settings [56-58].

#### Outcomes

The primary outcomes of this study are successful quit attempts and intention to quit. For this study, a successful quit attempt is defined as self-reported continuous abstinence from all forms of tobacco (smoked and SLT) for at least 3 consecutive months among participants who reported any tobacco use at baseline [59]. For 6-month follow-up, participants will be asked whether they have used any cigarettes, bidis, or other smoked products, and any SLT products (eg, gutka, khaini, pan with tobacco, and snuff) in the past 7 days, 30 days, and 3 months, and to report the date of last use [59,60]. Exclusive smokers will be classified as abstinent only if they report no tobacco use in the preceding 3 months, exclusive SLT users only if they report no SLT use, and dual users only if they report no use of either smoked or SLT products over this period. Any resumption of smoked or SLT product after at least 24 hours of abstinence will be considered a relapse, and participants will be classified as nonabstinent for the primary outcome. Intention to quit is defined as a current tobacco user reporting plans to quit within the next 3 months, 6 months, or later, reflecting varying levels of readiness to quit [59,60] (Table 2). The 3-month period used here aligns with intermediate outcome measures commonly applied in early-phase and digital cessation intervention trials [59,60]. The secondary outcome measures include acceptability, usability, and user engagement with the CARE interface.

**Table 2:** Summary of study outcomes.

Measures		Timelines		
		Baseline	After 3 months	6 months
Primary outcomes				
	Quit attempts	✓✓		✓
	Intention to quit	✓		✓
Secondary outcomes				
	Feasibility		✓	
	Acceptability		✓	
	User engagement		✓	
	Usability		✓	

### Data Analysis

Qualitative IDIs will guide the codevelopment of the CARE interface in phase 1 by informing design features, culturally tailored content, multilingual options, behavioral modules, as well as the development of survey items and outcome measures for phases 2 and 3. In phase 2, qualitative interviews will complement quantitative assessments by aiding interpretation of feasibility, acceptability, usability, and user engagement, while also identifying key implementation barriers and facilitators. In phase 3, qualitative insights will support interpretation of preliminary quantitative effectiveness outcomes, including quit attempts, intention to quit, and related psychosocial measures.

### Analysis of Qualitative Data

The qualitative data to be collected from the semistructured IDIs conducted with tobacco users and counselors will be transcribed verbatim and then translated into English. A framework approach will be used for analyzing the data [31,61]. The transcripts from IDIs will be independently analyzed by 2 researchers through an iterative process to develop themes and categories. For the development of the CARE conversational interface in phase 1, themes and categories will identify common patterns related to facilitators, barriers, and use of digital interventions among tobacco users. For phase 2, themes and subthemes will be categorized to comprehensively understand the feasibility, acceptability, usability, and engagement with the CARE. The results will be categorized into key focus areas as preset domains for feasibility, as suggested by Bowen et al [45]. All findings will be reported as per the COREQ (Consolidated Criteria for Reporting Qualitative Research) checklist [62].

### Analysis of Quantitative Data

The quantitative analysis will follow a structured analytic sequence. First, descriptive statistics (means, SDs, frequencies, and proportions) will summarize baseline characteristics and outcome distributions across study sites. Second, exploratory bivariate analyses will be conducted to examine crude associations between key exposures and outcomes; these will be interpreted descriptively. The primary cessation outcome (3-month continuous abstinence) and intention to quit (binary outcome) will be analyzed using mixed-effects logistic regression models with random intercepts for participant and study site. Time (baseline vs 6-month follow-up) will be included as a fixed effect. This approach allows estimation of within-participant change over time while accounting for between-site variability. Adjusted models will include predefined covariates such as age, sex, type of tobacco use, and baseline dependence level. All primary analyses will follow an intention-to-treat approach, with participants lost to follow-up classified as nonabstinent. For secondary and exploratory outcomes, analyses will be conducted using complete cases. Sensitivity analyses using multiple imputation may be undertaken to assess the robustness of findings, depending on the extent and pattern of missing data.

Continuous outcomes (eg, knowledge scores, self-efficacy scores, and decisional balance scores) will be analyzed using mixed-effects linear regression models with random intercepts for participant and study site. These models will estimate mean changes from baseline to follow-up while accounting for clustering and repeated measures. Paired 2-tailed *t* tests will be used only for preliminary descriptive comparisons and will not replace the primary mixed-effects analysis. Subgroup analyses will be conducted within the mixed-effects modeling framework by including interaction terms between time and subgroup variables (eg, tobacco type, sex, and place of residence). These analyses will be interpreted as exploratory. Mixed-effects models will be estimated using maximum likelihood methods with appropriate variance estimation. Model assumptions and goodness-of-fit will be assessed prior to interpretation. All statistical analyses will be performed using Stata version 17.0 (StataCorp LLC).

### Ethical Considerations

The study will adhere to ethical guidelines and principles, including obtaining informed consent from all participants, ensuring confidentiality and anonymity, and protecting participants’ rights throughout the research process. All study tools developed for data collection were reviewed and approved by the PHFI Institutional Ethics Committee (PHFI-IEC number: TRC-IEC/515/24/Exp/New) prior to data collection. Additionally, the study has been prospectively registered with the Clinical Trials Registry-India (CTRI number: CTRI/2024/11/076916), ensuring transparency and accountability in its conduct.

Given the intervention’s incorporation of a multilingual LLM with voice-based interaction, robust data protection and AI oversight measures have been integrated. The LLM app will be hosted on dedicated Amazon Web Services (AWS) infrastructure with access restricted to the authorized study team. Data will be encrypted in transit and at rest. All external or app-level access requires token-based authorization. Regular data backups and disaster recovery protocols are in place, and the platform follows Open Worldwide Application Security Project (OWASP) standards and Indian Computer Emergency Response Team (CERT-In) security guidelines to ensure secure and compliant operations. Consent management will be implemented directly within the front-end app. Participants can provide, review, or withdraw consent at any time, and upon withdrawal, their data, including historical interaction records, will be permanently deleted from the system. The app front-end will be accessible only to authorized end users through secure login credentials, ensuring participant confidentiality.

## Results

Recruitment of research staff across all study sites is expected to be completed by January 2026. Phase 1 (codevelopment) is scheduled from February to August 2026. Phase 2 (feasibility testing) will be conducted from September 2026 to August 2027, followed by phase 3 (effectiveness assessment) from September to November 2027. Data analysis and synthesis of the main findings are expected to be completed by the end of 2027. The study results are expected to be submitted for publication in a peer-reviewed, indexed scientific journal by December 2027.

## Discussion

### Anticipated Findings

This protocol outlines a single-group pre-post study aimed at codeveloping, implementing, and evaluating the CARE app, an AI-enabled, RAG-based LLM conversational interface designed to strengthen tobacco cessation in India. The anticipated findings are that integrating a culturally tailored, multilingual digital support with evidence-based behavioral frameworks will be feasible, acceptable, and usable among tobacco users attending TCCs. Through real-time personalized engagement, the CARE app is expected to enhance quit attempts, 3-month abstinence, and strengthen intention to quit over 6 months. This study will also generate evidence on the factors influencing engagement and user experience with AI-driven cessation interventions in real-world public health settings. However, given the absence of a concurrent control group, any observed improvements in cessation outcomes should be interpreted cautiously as preliminary indicators rather than definitive causal effects. Routine counseling at TCCs, secular changes in motivation, or external influences may also contribute to observed changes.

Existing Indian cessation efforts, including TCCs, national quitlines, and mCessation, have achieved moderate reach but limited sustained impact, with challenges such as stigma, accessibility issues, poor adherence [9,10], and high attrition, with only 3% to 8% of users accessing pharmacotherapy or counseling despite widespread quit intentions reported in GATS-2 [2]. Global meta-analyses indicate that conversational AI interventions can increase smoking cessation at 6-month follow-up (relative risk 1.29, 95% CI 1.13-1.46) compared to control group participants, though evidence remains limited by high heterogeneity and loss to follow-up [24,63]. Unlike most English language–based apps focused on smokers, the CARE app addresses critical gaps relevant to low- and middle-income countries’ (LMICs) contexts. It uniquely emphasizes codevelopment with users and providers and multilingual support for both smoker and SLT users in India by adopting the DISCOVER framework [31]. This approach aligns with the urgent need for context-specific, digital cessation tools in LMICs, where 80% of tobacco users reside and AI tools are scarce [1].

### Strengths and Limitations

The key strengths of this mixed methods study include the cocreation process with tobacco users and counselors, which ensured contextual and cultural relevance, and the use of validated psychometric instruments, including the Fagerstrom Test for Nicotine Dependence [52], Knowledge Score questionnaire [53], Process of Tobacco Cessation survey [32], Smoking Self-Efficacy questionnaire [54], and the Decisional Balance Scale [55]. The multisite design, encompassing both urban and rural TCCs across 3 locations, enhances participant diversity and improves the transferability of findings across service delivery contexts. In addition, the integration of a multilingual RAG–based LLM architecture, push notifications [64], gamification elements [65], craving tracking [66], and escalation pathways to human counselors is expected to enhance engagement and cultural tailoring, addressing limitations reported in earlier digital cessation interventions characterized by low user retention [4,67].

However, several limitations should be considered when interpreting the findings. The study uses a single-group pre-post design without a concurrent control arm, limits causal inference, and restricts attribution of observed changes solely to the CARE intervention. Outcomes are primarily self-reported, long-term follow-up beyond 6 months was not feasible, and generalizability is limited to treatment-seeking TCC users with access to smartphones. The findings may therefore not extend to individuals with severe mental health conditions or to populations with limited digital access. Additionally, biochemical verification of tobacco abstinence (eg, carbon monoxide breath testing or cotinine assays) was not conducted due to feasibility, cost, and logistical constraints across multiple study sites. As a result, cessation outcomes rely on self-reported measures and may be subject to recall or social desirability bias. Accordingly, results are interpreted conservatively, with greater emphasis placed on observed trends and relative changes over time rather than on precise estimates of abstinence.

### Future Directions

The findings of this study will inform RCTs comparing CARE-integrated TCCs with stand-alone TCCs or AI-only arms, assessing the dose-response effects of engagement on abstinence, cost-effectiveness, and integration with the NTQLs. Expanding voice recognition, incorporating long-term (>1 year) abstinence tracking, and adapting the system for Southeast Asia [67,68], along with subgroup analyses by tobacco type and region, will guide equitable scaling. Further expansion into non-TCCs, integration with pharmacotherapy, and evaluation of sustained impacts through biochemical verification can also strengthen implementation and support the World Health Organization Monitor, Protect, Offer, Warm, Enforce, and Raise (WHO MPOWER) strategies across SLT-prevalent countries [1,69].

### Conclusions

The CARE study represents an innovative pathway for leveraging conversational AI to enhance tobacco cessation in India. By combining user-centered design, behavioral theory, and multilingual AI support, the study aims to generate scalable insights into the feasibility, acceptability, usability, and preliminary effectiveness of digital tobacco cessation interventions. The anticipated results will help shape future implementation science and policy strategies advancing equitable, technology-enabled cessation services in LMICs.

## Appendix

Multimedia Appendix 1 Objectives, sample size, and data collection approach for the study.

## Abbreviations

AOR: adjusted odds ratio
ASR: automatic speech recognition
AWS: Amazon Web Services
BBCI: Dr. B. Borooah Cancer Institute
CARE: Comprehensive AI-Powered Conversational Interface to Quit Tobacco
CERT-In: Indian Computer Emergency Response Team
COREQ: Consolidated Criteria for Reporting Qualitative Research
CTRI: Clinical Trials Registry-India
DISCOVER: Designing, Developing, Evaluating, and Implementing a Smartphone-Delivered, Rule-Based Conversational Agen
GATS-2: Global Adult Tobacco Survey-2
HCP: health care professional
ICMR: Indian Council of Medical Research
IDI: in-depth interview
IIT-B: Indian Institute of Technology Bombay
LASI: Longitudinal Aging Study in India
LLM: large language model
LMIC: low- and middle-income countries
MPOWER: Monitor, Protect, Offer, Warm, Enforce, and Raise
NIMHANS: National Institute of Mental Health and Neurosciences
NTQL: National Tobacco Quitline Service
OWASP: Open Worldwide Application Security Project
PHFI: Public Health Foundation of India
IEC: Institutional Ethics Committee
RAG: retrieval-augmented generation
RCT: randomized controlled trial
SLT: smokeless tobacco
TCC: tobacco cessation center
TMC: Tata Memorial Centre
TTM: Transtheoretical Model
TTS: text-to-speech
WER: word error rate
WHO: World Health Organization
