# Supplementary material for: Codevelopment, Implementation, and Evaluation of an AI-powered Conversational Interface for Tobacco Cessation: Protocol for a Single-Group Pre-Post Study
Source: JMIR Res Protoc. 2026 Oct 7;15:e82264. doi: 10.2196/82264 (PMC13687822; doi:10.2196/82264)
Supplement: Supplementary file 1 [file resprot_v15i1e82264_app1.docx]

**Table S1.**

| **Objective** | **Sample Size** | **Target Participants** | **Data Collection Approach** |
| --- | --- | --- | --- |
| 1. To co-develop an AI-based conversational interface utilizing large language models to strengthen tobacco cessation behaviours at TCCs. | 48 | Tobacco users from TCCs (diverse in tobacco type, SES, and gender). | In-depth Interviews (16 participants per site × 3 sites). |
|  | 6 | TCC counsellors and healthcare providers (HCPs). | In-depth Interviews (2 participants per site × 3 sites). |
| **2.** To assess the feasibility, acceptability, usability, and user engagement with the AI-based conversational interface for tobacco users at TCCs. | 96  (32 participants per site) | Tobacco users currently using the AI-based conversational interface. | User experience data collection through semi-structured in-depth interviews.  Actual application usage patterns captured by the CARE interface.  Direct user feedback through structured Attitudinal Survey. |
| **3.** To evaluate the effectiveness of the AI-based conversational interface in facilitating successful quit attempts and increase intention to quit among tobacco users at TCCs. | 426  (142 participants per site) | The AI-based conversational interface from Project CARE will be evaluated with tobacco users to assess its effectiveness in supporting quit attempts (≥3 months abstinent) and increasing intention to quit (within 3–6 months). | Pre-post evaluation, surveys, and follow-up assessments **at baseline and** six-month follow-up. |
